# Supplementary material for: Acalculous Cholecystitis in a Young Adult with Scrub Typhus: A Case Report and Epidemiology of Scrub Typhus in the Maldives
Source: Trop Med Infect Dis. 2021 Dec 8;6(4):208. doi: 10.3390/tropicalmed6040208 (PMC8707333; doi:10.3390/tropicalmed6040208)
Supplement: Supplementary file 1 [file tropicalmed-06-00208-s001.zip › tropicalmed-1488805-supplementary.pdf]

Tropical Medicine and Infectious Disease

Supplementary Materials

Acalculous cholecystitis in a Young Adult with Scrub Typhus: A Case Report and Epidemiology of Scrub Typhus in the Maldives

Hisham Ahmed Imad [1,2,*], Aishath Azna Ali [3], Mariyam Nahuza [3], Rajan Gurung [3], Abdulla Ubaid [3], Aishath Maeesha [4], Sariu Ali Didi [4], Rajib Kumar Dey [4], Abdullah Isneen Hilmy [4,5], Aishath Hareera [6], Ibrahim Afzal [6], Wasin Matsee [7], Wang Nguitragool [1,8], Emi. E. Nakayama [2] and Tatsuo Shioda [2]

Mahidol Vivax Research Unit, Faculty of Tropical Medicine, Mahidol University, Bangkok 10400, Thailand; wang.ngu@mahidol.edu

2 Department of Viral Infections, Research Institute for Microbial Diseases, Osaka University, Suita 565-0871, Osaka, Japan; emien@biken.osaka-u.ac.jp (E.E.N.); shioda@biken.osaka-u.ac.jp (T.S.)

3 Department of Surgery, Indira Gandhi Memorial Hospital, Malé 20002, Maldives; dr.azna@igmh.gov.mv (A.A.A.); dr.mariyamnahuza@igmh.gov.mv (M.N.); dr.rajangurung@igmh.gov.mv (R.G.); dr.ubaid@igmh.gov.mv (A.U.)

4 Department of Medicine, Indira Gandhi Memorial Hospital, Malé 20002, Maldives; dr.aishathmaesha@igmh.gov.mv (A.M.); sariualididi@igmh.gov.mv (S.A.D.); dey@igmh.gov.mv (R.K.D.); hlmabd001@myuct.ac.za (A.I.H.)

5 Gastrointestinal Unit, Department of Medicine, Groote Schuur Hospital, University of Cape Town, Cape Town 7935, South Africa

6 Health Protection Agency, Ministry of Public Health, Malé 20002, Maldives; hareera@health.gov.mv (A.H.); afzal@health.gov.mv (I.A.)

7 Department of Clinical Tropical Medicine, Faculty of Tropical Medicine, Mahidol University, Bangkok 10400, Thailand; wasin.mat@mahidol.edu

8 Department of Molecular Tropical Medicine and Genetics, Faculty of Tropical Medicine, Mahidol University, Bangkok 10400, Thailand

* Correspondence: hishamahmed.ima@mahidol.ac.th or imad@biken.osaka-u.ac.jp; Tel.: +66-631501402

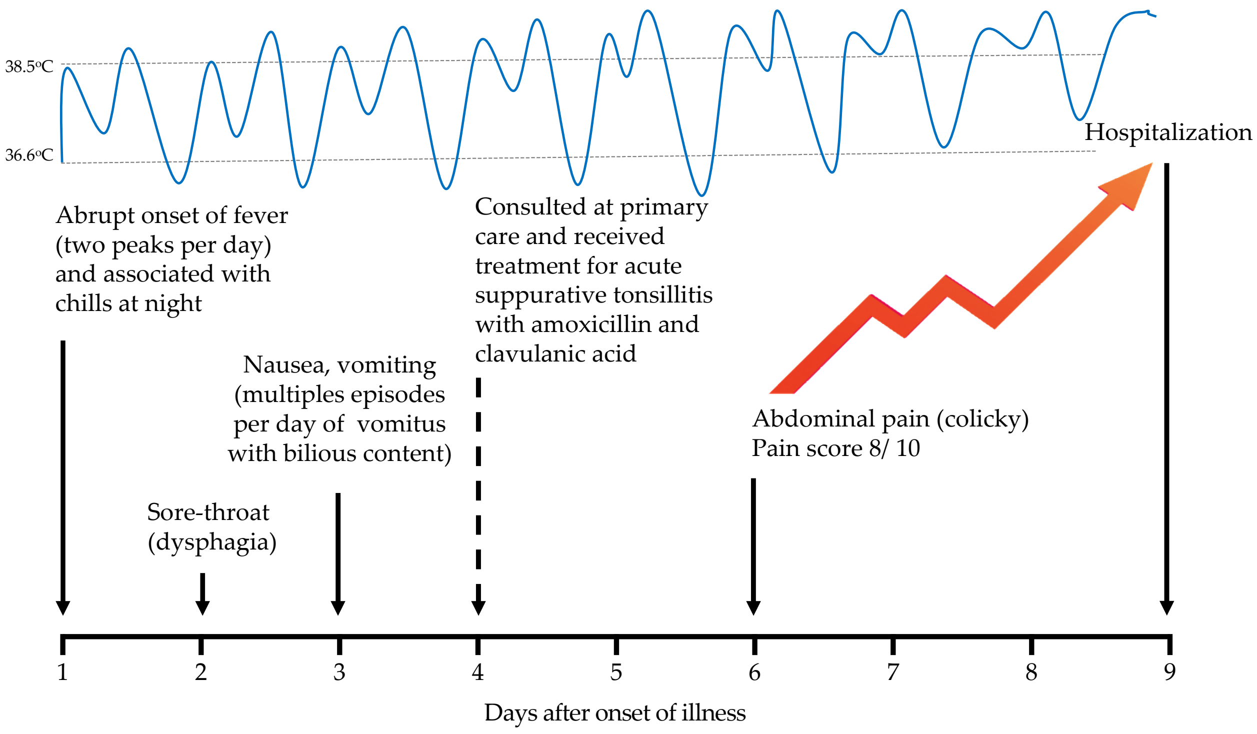

Temperature depicted is not the actual reading, but graphically presented to demonstrate the fever pattern described by the patient

Figure S1. Timeline depicting the clinical course prior to hospitalization

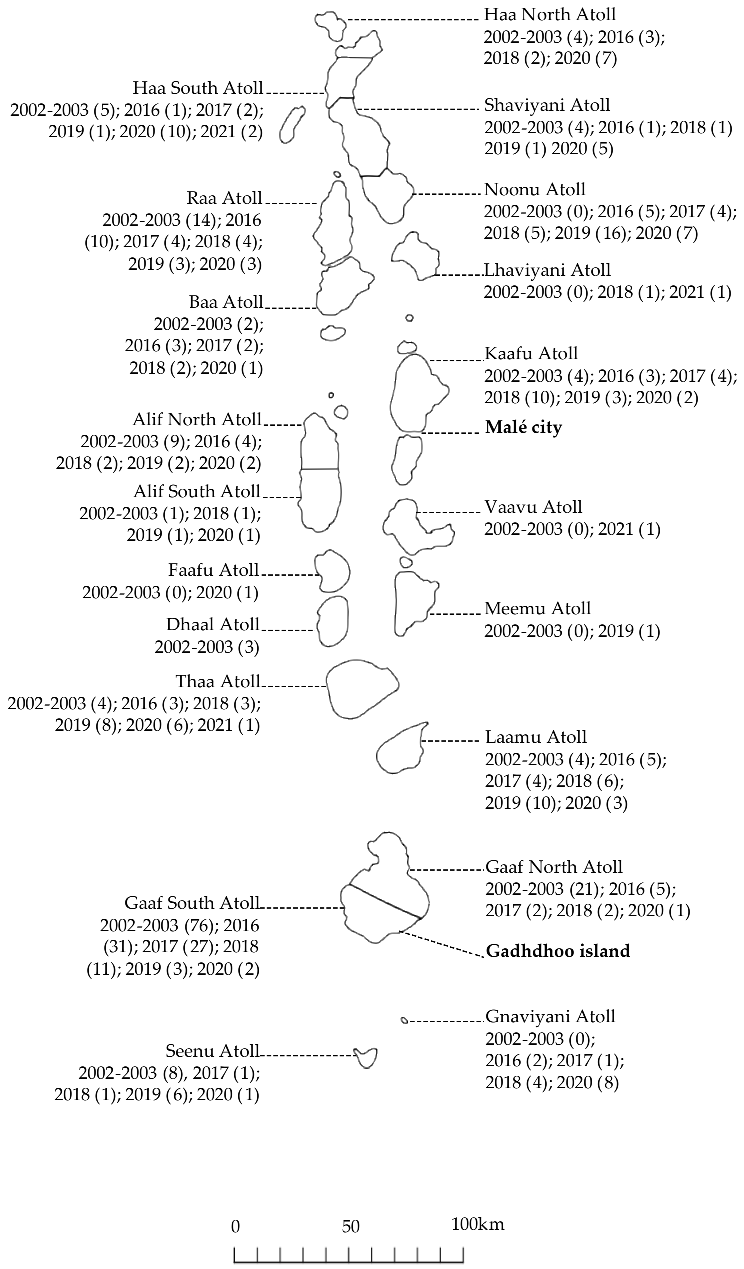

The data shown are the total number of reported cases, in parentheses by year. The 2002-2003 data were obtained from Lewis et. al. Scrub typhus re-emergence in the Maldives, Emerging Infectious Diseases 2003.

Figure S2. Past and recent, epidemiology of scrub typhus in atolls of the Maldives

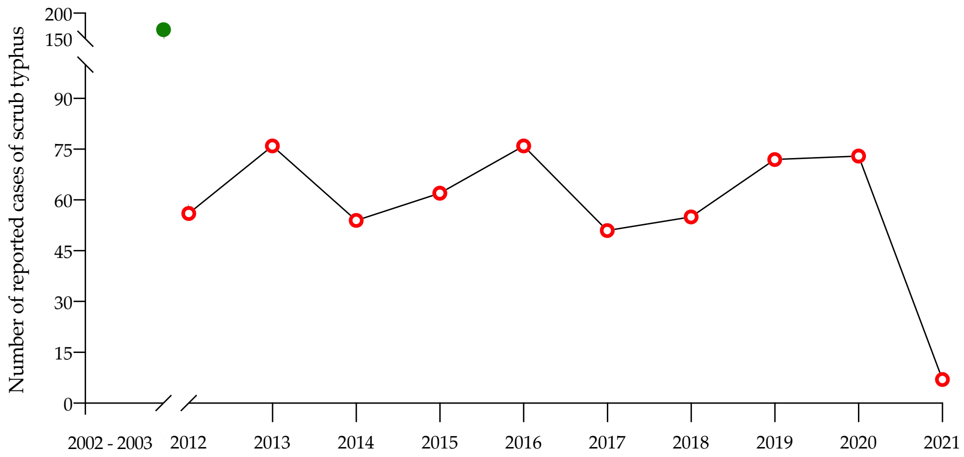

The graph shows the reported cases of scrub typhus to the Health Protection Agency, Ministry of Health, from 2012 through 2021. Shown in green is when scrub typhus re-emerged in the Maldives, sixty decades later since the first reporting in 1942.

Figure S3. Two decades of sentinel reporting of scrub typhus in the Maldives.

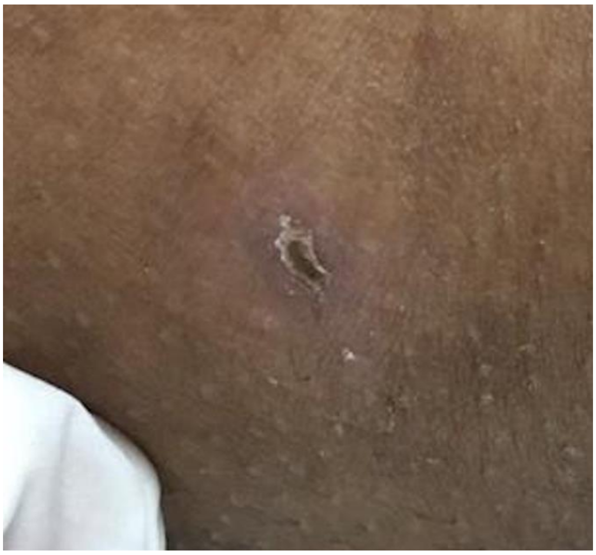

Figure S4. Eschar lesion on the medial aspect of thigh.

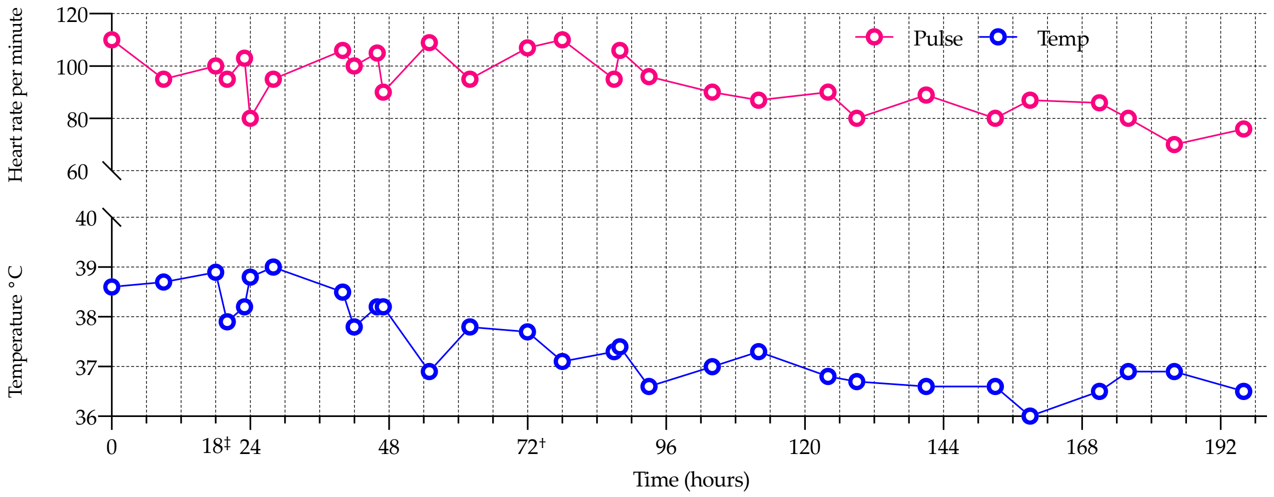

The graph shows the heart rate and temperature measured during the course of the hospitalization, ‡ Time doxycycline was initiated, † Time fever subsided and the shaded area in green is the fever clearance time after administration of doxycycline.

Figure S5. Clinical course during hospitalization.
